# Exploring the roles of phytobiotics in relieving the impacts of *Edwardsiella tarda* infection on fish: a mini-review

**DOI:** 10.3389/fvets.2023.1149514

**Published:** 2023-07-05

**Authors:** Khang Wen Goh, Zulhisyam Abdul Kari, Wendy Wee, Nik Nur Azwanida Zakaria, Mohammad Mijanur Rahman, Muhammad Anamul Kabir, Noor Khalidah Abdul Hamid, Albaris B. Tahiluddin, Ahmad Syazni Kamarudin, Guillermo Téllez–Isaías, Lee Seong Wei

**Affiliations:** ^1^Faculty of Data Science and Information Technology, INTI International University, Nilai, Malaysia; ^2^Department of Agricultural Science, Faculty of Agro-Based Industry, Universiti Malaysia Kelantan, Jeli, Kelantan, Malaysia; ^3^Advanced Livestock and Aquaculture Research Group, Faculty of Agro-Based Industry, Universiti Malaysia Kelantan, Jeli, Kelantan, Malaysia; ^4^Center of Fundamental and Continuing Education, Universiti Malaysia Terengganu, Kuala Nerus, Terengganu, Malaysia; ^5^Department of Agro-Based Industry, Faculty of Agro-Based Industry, Universiti Malaysia Kelantan, Jeli, Kelantan, Malaysia; ^6^Department of Aquaculture, Sylhet Agricultural University, Sylhet, Bangladesh; ^7^School of Biological Sciences, Universiti Sains Malaysia, Minden, Pulau Pinang, Malaysia; ^8^College of Fisheries, Mindanao State University-Tawi-Tawi College of Technology and Oceanography, Bongao, Tawi-Tawi, Philippines; ^9^School of Animal Science, Aquatic Science and Environment, Faculty of Bioresources and Food Industry, Universiti Sultan Zainal Abidin (UniSZA), Besut Campus, Besut, Terengganu, Malaysia; ^10^Department of Poultry Science, University of Arkansas, Fayetteville, AR, United States

**Keywords:** phytobiotics, *Edwardsiella tarda*, medicinal herbs, disease tolerance, antibacterial, sustainable aquaculture

## Abstract

Edwardsiellosis caused by *Edwardsiella tarda* resulted in significant economic losses in aquaculture operations worldwide. This disease could infect a wide range of hosts, including freshwater, brackish water, and marine aquatic animals. Currently, antibiotics and vaccines are being used as prophylactic agents to overcome Edwardsiellosis in aquaculture. However, application of antibiotics has led to antibiotic resistance among pathogenic bacteria, and the antibiotic residues pose a threat to public health. Meanwhile, the use of vaccines to combat Edwardsiellosis requires intensive labor work and high costs. Thus, phytobiotics were attempted to be used as antimicrobial agents to minimize the impact of Edwardsiellosis in aquaculture. These phytobiotics may also provide farmers with new options to manage aquaculture species' health. The impact of Edwardsiellosis in aquaculture worldwide was elaborated on and highlighted in this review study, as well as the recent application of phytobiotics in aquaculture and the status of vaccines to combat Edwardsiellosis. This review also focuses on the potential of phytobiotics in improving aquatic animal growth performance, enhancing immune system function, and stimulating disease resistance.

## Introduction

Nowadays, food security is a major concern throughout the world. Aquaculture can provide a reliable and affordable protein source for human consumption (1). This statement was supported by the data recorded that fish consumption per capita was 9 kg annually in 1961. The value increased rapidly to 20.5 kg in 2018 (2). The aquaculture industry is gearing up to fulfill the increasing demand for fish protein in the market. However, issues such as high stocking density and water quality have led to disease outbreaks (3), resulting in low production, poor growth performance, and a high mortality rate. Additionally, these issues also result in high operational costs and food insecurity and affect investors' income (4). Fish mortality rates due to disease outbreaks as high as 50% reported in developing countries have led some fish farmers to abandon their aquaculture operations. Based on the World Bank report, the disease outbreak caused approximately USD 6 billion in economic losses annually (5). The outbreaks may be due to pathogenic bacteria and stressful environmental conditions. Consequently, fish farmers had no option but to continue using antibiotics as a treatment against disease outbreaks (6).

*Edwardsiella tarda* was first reported in the literature by a Japanese scientist in 1962. This Gram-negative anaerobic facultative Brevibacterium infects a huge range of hosts, such as aquatic animals, amphibians, reptiles, and mammals throughout the world (7). *E. tarda* has infected various aquaculture species and has led to a huge economic loss (8, 9). Hemolysin is an important virulence factor of *E. tarda* that causes septicemia in the host (10). Other virulence factors that are responsible and involved in the infection process are catalase (11), Translocation and Assembly Module (Tam) (12), DNA-binding protein from starved cells (Dps) (13), undecaprenyl phosphate gylcosyltransferase (WcaJ) (14), and superoxide dismutase (15). The pathogenicity and virulence of *E. tarda* were reported due to the presence of virulence genes in the bacterium, namely, vibrioferrin synthesis (pvsA), sensor protein (qseC), chondroitinase (cds1), AHL-synthase (edwI), and DNA Gyrase (gyrB) (8, 16, 17).

**Figure 1:**
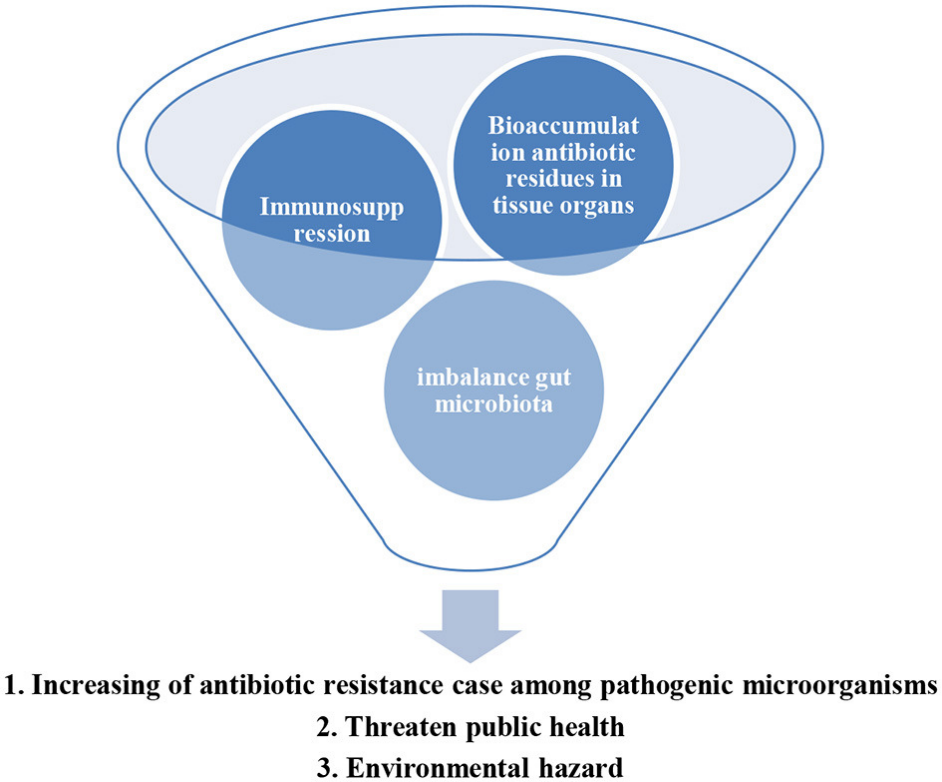
The impacts of antibiotics used in aquaculture.

The symptoms of Edwardsiellosis in infected fish species, such as hybrid snakehead (*Channa maculate* ♀ × *Channa argus* ♂) and grass carp (*Ctenopharyngodon idella*), are exophthalmia, hernia, internal organ damage (18), pigment loss, swollen anus, and enlarged kidney (19). Other symptoms reported in the literature are ascites and internal organ swelling (20). At present, antibiotics are used to lessen the impact of Edwardsiellosis on aquaculture species. However, the excessive use of antibiotics has led to an increment in antibiotic resistance cases against pathogenic *E. tarda* (21–24). For instance, *E. tarda* isolated from Siamese crocodile was found to be highly resistant to erythromycin, tetracycline, and oxytetracycline (25). Turbot farming in China was reported to rely on antibiotics and chemicals to combat Edwardsiellosis infection (26). In Korea, *E. tarda* isolated from farmed marine fishes was reported to be resistant to various antibiotics, such as streptomycin, cefaclor, lincomycin, penicillin, erythromycin, and rifampin (27). The application of antibiotics in aquaculture can control bacterial infection in the short term. However, adverse effects of using antibiotics as treatment include bioaccumulation of the antibiotic residues in aquatic animal tissues and organs, immunosuppression, and imbalance of gut microbiota (Figure 1) (28). The plasmid in *E. tarda* was found to carry antibiotic-resistance genes against multi-antibiotics (29). The application of antibiotics in aquaculture has led to the contamination of antibiotic residues in the human food chain (24). Furthermore, over usage of antibiotics in aquaculture can accelerate the emergence of multi-antibiotic-resistant pathogenic bacteria that can adversely affect public health (30). Therefore, there is a need to find alternative antimicrobial agents to control Edwardsiellosis in aquaculture to reduce overreliance on chemicals and antibiotics. This review discusses and summarizes the impact of Edwardsiellosis due to *E. tarda*, the application of phytobiotics in aquaculture, the status of the Edwardsiellosis vaccine, and the roles of phytobiotics in improving growth performance, enhancing the immune system, and stimulating disease resistance against *E. tarda*.

## Phytobiotics and their bioactive compound roles

Phytobiotics are plant-based derivatives that have beneficial effects on organisms. The bioactive compounds are responsible for the biological activities of phytobiotics, such as alkaloids, carotenoids, and phenolic compounds (31). The biological activities of phytobiotics can be anti-inflammatory, antimicrobial, antioxidant, and others. Generally, phytobiotics play an important role in promoting the growth of gut microbiota, increasing feed efficiency, and activating immune-related genes to enhance the immune system of fish (31). For example, Brown alga, *Ecklonia cava*, was found to promote the growth of probiotic lactic acid bacteria (LAB) in zebrafish and modulate the immune system of the fish against Edwardsiellosis infection (32).

## Impacts of edwardsiellosis due to *E. tarda* in aquaculture

**Figure 2:**
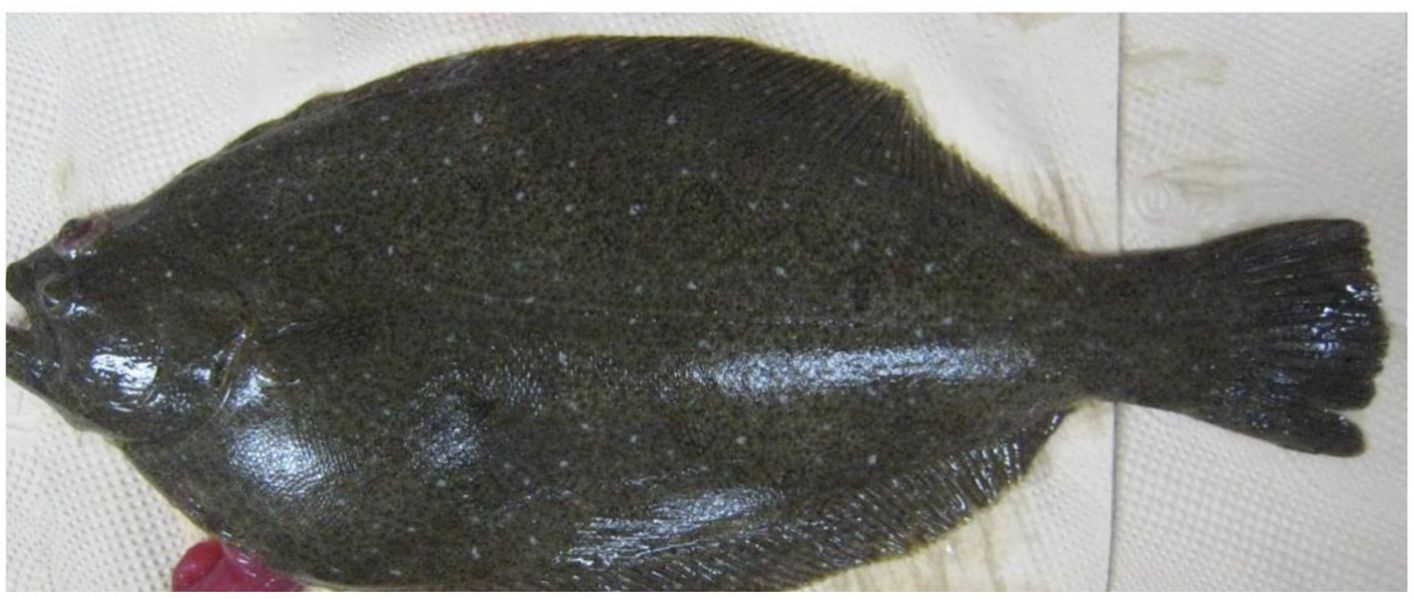
Edwardsiellosis infection in olive flounder, *Paralichthys olivaceus* (9).

*E. tarda* is an important disease-causing bacterium in aquaculture (33). This bacterium is under the genus of *Edwardsiella*. There are another four pathogenic bacteria under similar genera, namely, *Edwardsiella. anguillarum* (34), *Edwardsiella piscicida* (35), *Edwardsiella ictaluri*, and *Edwardsiella hoshinae*. *E. tarda* is a short rod-shaped Gram-negative bacterium with a diameter of 1–3 μm in length (36, 37). Based on the phenotypes, *E. tarda* can be divided into two groups, namely, typical and atypical (38). Typical and atypical groups are referred to as motile and non-motile *E. tarda*, respectively. The bacterium can be grouped into four serotypes (i.e., A, B, C, and D). The serotype grouping is based on the agglutination of the bacterium with specific antisera to identify variants of somatic (O) and flagella (H) antigens. This bacterium is responsible for Edwardsiellosis disease outbreaks in many fish farming. For instance, the Edwardsiellosis outbreak was reported in carp species, such as crucian carp in Japan (39) and grass carp in India (19, 40). Besides carp, Edwardsiellosis also infected Japanese eels in Fujian Province in China (41), giant mottled eels in China (42), and Japanese eels in South Korea (43). Edwardsiellosis has caused high mortality of hybrid snakeheads in China (18). Many cases of Edwardsiellosis outbreaks were recorded in olive flounder (Figure 2) and Japanese flounder farms in China (44–46). Furthermore, Edwardsiellosis has infected Chinook salmon in the US (47), Sharpsnout seabreams in Greece (48), Rainbow trout in Korea (49), Dabry sturgeon in China (50), Yellow catfish in China (51), Black rockfish in China (52), Chinese tongue sole (53) in China, Seahorse (54) in China, Siamese crocodile in Hainan and China (25), and Chinese soft-shelled turtle in China (55). *E. tarda* caused ascites disease in juvenile turbot (*Scophtalmus maximus*), which led to high mortality (30–50%) (56). In some cases, ascites in turbot have led to massive mortality, as high as 90% (41). As a result, Edwardsiellosis poses a significant threat to turbot farming, especially in producing seeds. Besides, *E. tarda* is also responsible for gangrene in fish, red disease in eels, emphysematous putrefactive disease in catfish (36), and fatal septicemia in newly hatched farmed crocodiles (57, 58). In the US, Edwardsiellosis is a major disease in farmed channel catfish, *Ictalurus punctatus*, and farmed barramundi, *Lates calcarifer* (59). Furthermore, Edwardsiellosis is a well-known disease in Egypt during summer that has caused huge mortality in Nile tilapia (60). The impact of Edwardsiellosis infection in fish organs is shown in Table 1 (61). This disease can spread through contaminated feed, water, or intestinal mucosa, and a poor environment, such as the presence of high organic, poor water quality, and high temperature can trigger Edwardsiellosis infection in fish (61). Overall, Edwardsiellosis is an important disease in aquaculture that has a huge economic impact. Edwardsiellosis-causing bacterium, *E. tarda*, can adapt to a wide range of environments and infect various hosts resulting in high mortality. Edwardsiellosis outbreak devastates many fish farmers causing them to sometimes end the farm's operation. In addition, investors also lose their income and many workers become jobless.

**Table 1:** The impacts of Edwardsiellosis on fish organs.

**Fish organs**	**Clinical signs**
Scale/body	Loss of pigmentation
Fin/skin	Hemorrhage
Eyes	Exophthalmia/opacity
Abdominal space	Bloody ascites
Liver, spleen, and kidney	Congestion

## Application of phytobiotics in aquaculture

Phytobiotics are referred to as any plant-based products that have antimicrobial activity (31, 62) and antioxidant capacity, can promote growth performance (63), enhance the immune system (64), stimulate disease resistance, and mitigate stress. There are some phytobiotics have been approved by the U.S. Food and Drug Administration (FDA) to be used in animal production (65) such as cottonseed meal and rice hull. However, both phytobiotics cannot be applied more than 20% in the feed formulation. Different phytobiotics vary in their modes of action depending on the bioactive component. Generally, phytobiotics can modulate gut microbiota and change the membrane permeability of pathogenic microorganisms. However, the effectiveness of phytobiotics can be influenced by many factors, such as storage conditions, post-harvesting processing, geographical locations, and plant species (66, 67).

**Figure 3:**
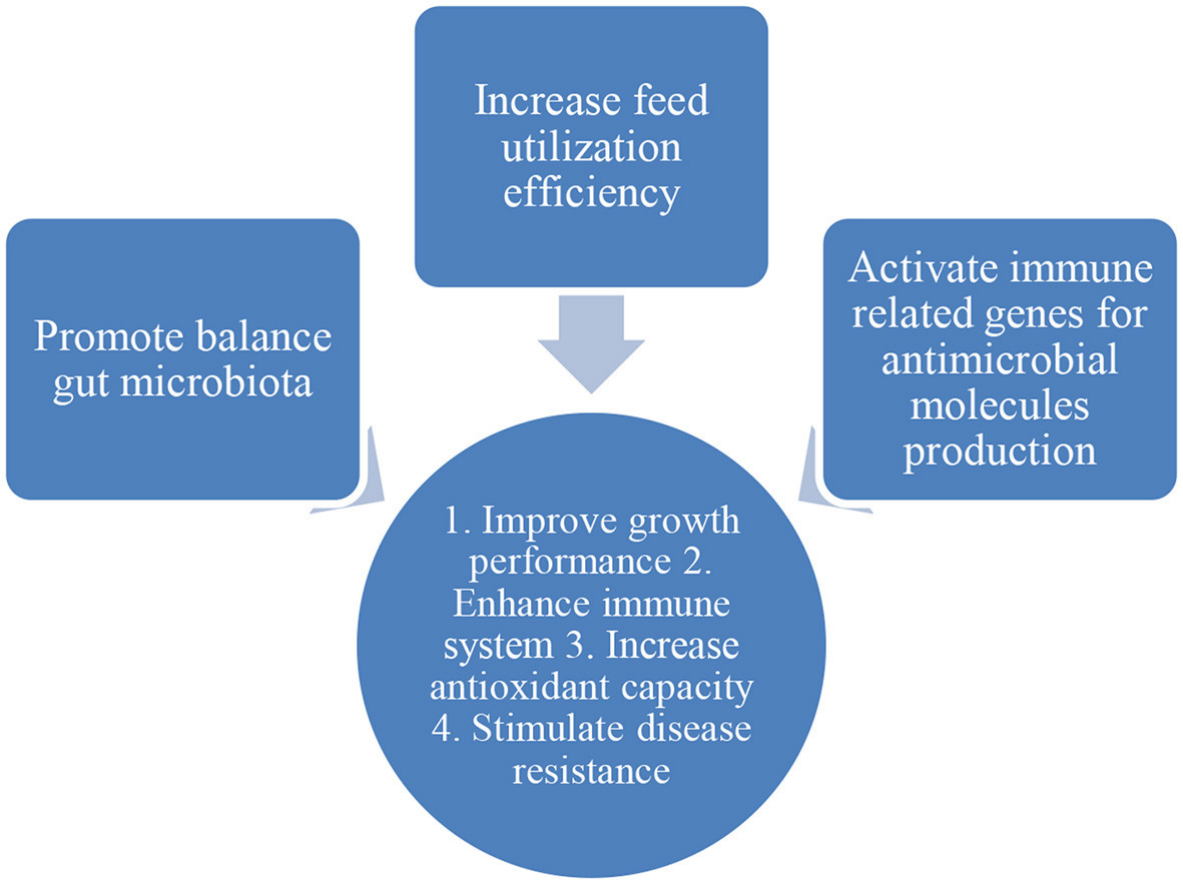
Mode of action of phytobiotics.

The potential of phytobiotics (e.g., essential oil, plant leaves, flowers, and alcoholic extracts) usage in aquaculture was widely documented (Table 2). Phytobiotics were used as feed additives in aquaculture, and some were used as a solution and medicated through bathing treatment. The exposure period of aquaculture species to phytobiotics ranges from 14 to 60 days. Generally, the modes of action of phytobiotics are to promote the growth of gut microbiota, increase feed utilization efficiency, and activate immune-related genes to produce antimicrobial production (68–72) (Figure 3). Based on the phytobiotics' mode of action, the beneficial effects of phytobiotics on aquaculture species include enhancing the immune system, increasing antioxidant activity, improving growth performance, and stimulating disease resistance of aquaculture species. Besides, phytobiotics can also be used as alternate commercial antibiotics, acting as antimicrobial agents and mitigating abiotic stress such as ammonia.

**Table 2:** Phytobiotics used in aquaculture.

**Species**	**Phytobiotics/Bioactive compounds**	**Dose**	**Duration**	**Effects**	**References**
Nile tilapia, *O. niloticus*	Volatile oils of thyme, red thyme, and pepper rosemary/terpenes, terpenoids	1.2 g/kg of feed	20 days	Enhance immune system; Replace antibiotic enrofloxacin; High antioxidant activity; Stimulate disease resistance against *A. hydrophila*	(28)
Nile tilapia, *O. niloticus*	*Bougainvillea glabra* leaf/tannin, alkaloids	4.5%/kg of feed	30 days	Improve growth performance; Stimulate disease resistance against *Enterococcus faecalis*	(73)
Striped catfish, *Pangasianodon hypophthalmus*	Milk thistle, *Silybum* marianum/polysaccharides	0.1–0.3%/kg of feed	60 days	Improve growth performance; Enhance immune system; Increase antioxidant capacity	(74)
Caspian roach, *Rutilus caspicus*	Essential oil of savory, *Satureja hortensis*/terpenes, terpenoids	200 mg/kg of feed	60 days	Improve growth performance Stimulate stress resistance against salinity	(75)
African catfish, *Clarias gariepinus* (B.)	Leaf of clove basil, *Ocimum gratissimum*/tannin, alkaloids	12 g/kg of feed	84 days	Improve growth performance; Enhance immune system; Increase antioxidant capacity; Stimulate disease resistance against *Listeria monocytogenes*	(76)
Nile tilapia, *O. niloticus*	Probiotic (*Bacillus subtilis* + *Bacillus licheniformis*) + *Yucca schidigera* solution extract/Polysaccharides	5 × 10^10^ cfu/g + 0.11 ml/m^3^	14 days	Enhance immune system; Increase antioxidant capacity; Stimulate stress resistance against ammonia	(77)
Nile tilapia, *O. niloticus*	Commercial seaweed liquid extract (TrueAlgaeMax, TAM)/polysaccharides	50–200 ml/m^3^	70 days	Improve growth performance; Enhance immune system; Stimulate disease resistance against *A. hydrophila*	(78)
Nile tilapia, *O. niloticus*	Alcoholic extract of *Artemisia annua*/tannin, alkaloids	0.1–0.5% per kg of feed	30 days	Improve growth performance; Enhance immune system; Promote the growth of beneficial gut microbiota	(79)
Common carp, *C. carpio* L. fingerling	Oregano essential oil/terpenes, terpenoids	5–20 g/kg of feed	56 days	Improve growth performance	(80)
Great sturgeon, *Huso huso*	Rosemary essential oil/terpenes, terpenoids	0.01–2% per kg of feed	56 days	Improve growth performance	(81)
Red drum, *Sciaenops ocellatus*	*Ocimum americanum* essential oil/terpenes, terpenoids	0.25–2g/kg of feed	49 days	Improve growth performance; Enhance immune system	(82)
Nile tilapia, *O. niloticus*	β-Glucan with/without probiotic *B. coagulans*	β-Glucan – 0.1 g/kg of feed *B. coagulans* – 1–2 g/kg of fish	98 days	Improve growth performance; Enhance immune system; Increase antioxidant capacity	(83)
Nile tilapia, *O. niloticus*	Miswak (*S. persica*) leaf/salvadorine, trimethylamine, tannins, flavonoids, saponins, sulfur	2.5–10 g/kg of feed	56 days	Improve growth performance; Enhance immune system; Increase antioxidant capacity; Stimulate disease resistance to *A. hydrophila*	(84)
Nile tilapia, *O. niloticus*	Brown seaweed, *S. aquifolium/*polysaccharides	50–200 g/kg of feed	56 days	Improve growth performance; Enhance immune system; Increase antioxidant capacity; promote the growth of gut microbiota	(85)
Nile tilapia, *O. niloticus*	*N. oculata*/essential vitamins and polysaccharides	5-15% of diet	64 days	Improve growth performance; Enhance immune system; stimulate disease resistance against *Aeromonas veronii*	(86)
Common carp, *C. carpio*	*O. vulgare* essential oil	0.5–1% of diet	30 days	Relieve oxidative stress due to cypermethrin	(87)
Nile tilapia, *O. niloticus*	Thymol Thymol + chitosan nanoparticle	0.5 g/kg of diet 0.5 g/kg of diet + 5 g/kg of diet		Promote feed utilization, antioxidant, and health status	(88)
Nile tilapia, *O. Niloticus*	Rosemary/carnosol and rosmanol	0.5% of diet		Relieve suppression of aflatoxin on growth and feed utilization	(89)

β-Glucan is a commercial polysaccharide that can be used as an immunostimulant. It was reported to increase the binding activity of receptors with natural killer cells and neutrophils (83). Hence, β-glucan can enhance the immune system. Many studies revealed that β-glucan could improve the immune system in aquatic animals, such as *Oreochromis niloticus* (83), *Litopenaeus vannamei* (90), *Oncorhynchus mykiss* (91), *Lutjanus peru* (92), *Cyprinus carpio* (93), and *Trachinotus ovatus* (94). In addition, a combination of probiotic *Bacillus coagulans* and β-glucan can perform a synergistic effect to enhance the immune system of *O. niloticus* (83). Dietary Miswak (*Salvadora persica*) leaf in Nile tilapia, *O. niloticus*, was found to have beneficial effects, such as growth performance improvement, immune system enhancement, antioxidant increment, and *Aeromonas hydrophila* disease resistance stimulation (84). The beneficial effects were linked to the bioactive compounds present in the phytobiotic, such as alkaloids comprising salvadorine, trimethylamine, tannins, flavonoids, saponins, and sulfur (95). These bioactive compounds have been stated to promote feed consumption, relieve stress, and act as immunostimulants (84). Besides, dietary of Miswak root was also reported to promote an immune system of common carp (96) and tilapia (97). Nile tilapia, *O. niloticus* that received brown seaweed *Sargassum aquifolium* in diet performed significantly better in growth and health (85). Polysaccharides, the bioactive compounds that were present in brown seaweed, were responsible for the positive response in Nile tilapia, *O. niloticus*. The polysaccharides can promote the growth of gut microbiota (32) and activate gene-related antimicrobial production molecules (98). Thus, dietary brown seaweed can promote feed utilization efficiency and the health status of Nile tilapia.

The potential of microalga, *Nannochloropsis oculata*, as a phytobiotic was revealed in the study of Abdelghany et al. (86). Dietary *N. oculata* at doses of 5–15% of the diet was found to promote the growth and health status of Nile tilapia, *O. niloticus*. Microalgae are widely used in aquaculture as they are rich in essential amino acids. Besides, they also carry bioactive compounds such as essential vitamins and polysaccharides that can fortify health status and promote growth performance (108). However, the application of *N. oculata* as a feed additive at higher doses may lead to disruption of nutrient digestion activity (109), and the presence of complexed non-starched polysaccharides, such as pectins, gums, cellulose, and hemicelluloses, can reduce nutrient absorption in the fish digestive system (110). Therefore, *N. oculata* must use in the optimal range to avoid adverse impacts on the fish. A diet of rosemary (*Rosmarinus officinalis*) was found to relieve aflatoxin B1-suppressed growth and feed utilization in Nile tilapia (89). Rosemary is a well-known herb for its high antioxidant activity (111). Polyphenol compounds are the main bioactive compounds in rosemary that are responsible for their antioxidant property (112). Carnosol and rosmanol are two bioactive compounds that are present in rosemary. These bioactive compounds can enhance nutrient digestibility and inhibit pathogenic bacteria in the intestine (113).

Dietary *Origanum vulgare* essential oil at the dose of 0.5–1% of the diet was reported to relieve oxidative stress due to the presence of insecticide, cypermethrin, in common carp, *C. carpio* (87). The bioactive compounds, which are present in the essential oil, such as carvacrol, thymol, cymene, and terpinene, are able to increase the antioxidative capacity of fish (80, 114). Besides, *O. vulgare* essential oil was also reported to mitigate oxidative stress due to carbon tetrachloride (115), gentamycin (116), and paraquat (117) in rats. Thymol is another phytobiotic that was reported to have a positive impact on aquaculture species. However, the application of thymol alone showed no significant impact on the growth performance in rainbow trout (118), channel catfish (119), and Nile tilapia (120). However, thymol, in combination with other prophylactic agents, was found to promote the growth performance of fish. For instance, a dietary combination of thymol and carvacrol can help to promote the growth performance of European sturgeons (121) and gilthead seabreams (122). In addition, a dietary combination of chitosan nanoparticles and thymol was found to promote the growth and health status of Nile tilapia (88).

## Phytobiotics vs. commercially developed vaccines against edwardsiellosis due to *E. tarda*

Several studies have shown the potential use of vaccines in aquaculture against edwardsiellosis (Table 3). for instance, Castro et al. (123) and Lan et al. (124) reported that an effective edwardsiellosis vaccine has been developed for turbot, *Sauertylenchus maxinus*. inactivated *E. tarda* vaccine also has been shown to stimulate immune response effectively in zebrafish (125), flounder (46, 126), turbot (127), tilapia, and *Oreochromis mossambicus* (128), and japanese flounder, *P. olivaceus* (129). the studies have used different immunogens, such as whole cell, live cells extract, outer membrane protein (130), and attenuated *E. tarda*, to stimulate the immune response in fish against edwardsiellosis. although the application of vaccines can control edwardsiellosis effectively, issues, such as cost, huge labor requirements, and species-specific usage, have limited its use. besides, the application of live vaccines has legal objections in many countries. meanwhile, selective breeding has been proposed as a method to improve the genetics of aquaculture species to counter the disease resistance issue against *E. tarda* (13, 131, 132).

**Table 3:** Phytobiotics used to mitigate *E. tarda* impacts on aquatic animals.

**Species**	**Phytobiotics/bioactive compounds**	**Dose**	**Duration**	**Effects**	**References**
Rockfish, *Sebastes schlegelii*	Sponge seaweed, *Codium fragile*, derived sulfated polysaccharides/Sulfated polysaccharides	0.1–1%/kg feed	14–28 days	Enhance immune system	(72)
African catfish, *C. gariepinus*	*M. scaber* leaves extract/eugenol and gallic acid	6 g/kg of feed	56 days	Improve growth performance; Enhance immune system	(99)
*L. rohita*	Green alga, *C. aerea* extract/polysaccharides	50 mg/kg of feed	28 days	Stimulate disease resistance against Edwardsiellosis	(98)
*C. gariepinus*	Cassic acid	1–5 mg/kg feed	30 days	Improve growth performance; Stimulate disease resistance against Edwardsiellosis	(100)
Catla, *C. catla*	*Astragalus* polysaccharides	200–300 mg/kg of feed	56 days	Improve growth performance; Enhance immune system; Stimulate disease resistance against Edwardsiellosis	(101)
Zebrafish	Enzymatic extract of the brown alga, *E. cava*/polysaccharides	1%/kg of feed	21 days	Act as prebiotic; Promote the growth of probiotic in fish; Stimulate disease resistance against Edwardsiellosis	(32)
*C. gariepinus*	Methanol extract of apple mangrove, *S. caseolaris*/tannin, alkaloids	1.59–3.17 g/kg of feed	28 days	Improve growth performance; Stimulate disease resistance against Edwardsiellosis	(102)
Mozambique tilapia, *O. mossambicus*	Citrus limon peels essential oil/terpenes, terpenoids	0.5–1% of feed	60 days	Improve growth performance; Enhance immune system	(103)
Rock bream, *Oplegnathus fasciatus*	Leaves of Baical skullcap, *Scutellaria baicalensis* + probiotic *Lactobacillus sakei*/tannin, alkaloids	1% + 1%/kg feed	42 days	Enhance immune system; Stimulate disease resistance against Edwardsiellosis	(104)
Zebrafish larvae	Nanoscale β-glucan from oat	Bathing 100–500 μg/ml	3 days	Enhance immune system; Stimulate disease resistance against Edwardsiellosis	(105)
Korean rockfish, *Sebastes schelgeli*	Citrus by-product; fermented citrus by-product/flavonoid, coumarin, limonene	Equivalent to 100 mg ascorbic acid/kg feed	91 days	Improve growth performance; Stimulate disease resistance against Edwardsiellosis	(106)
Olive flounder, *P. olivaceus*	Ethanolic lacquer tree, *Rhus verniciflua* Stokes (RVS)/tannin, alkaloids	30–300 mg/kg of feed	14–70 days	Stimulate disease resistance against Edwardsiellosis	(107)

Phytobiotics have been shown to stimulate disease resistance in various aquaculture species. For example, a recent study by Ahmadifar et al. (68) has claimed that Cornelian cherry (*Cornus mas L*.) fruit extract can stimulate disease resistance in common carp, *C. carpio*, against *A. hydrophila*. Meanwhile, studies found that phytobiotics can stimulate disease resistance in aquatic animals (69–71). Sulfated polysaccharides from sponge seaweed (72) and *Astragalus* (101) were found to enhance the disease resistance of both freshwater and marine aquaculture species against Edwardsiellosis (Table 2). Sulfated polysaccharides are referred to as anionic polysaccharides that carry sulfates (133). These sulfated polysaccharides have medicinal benefits, such as antibacterial, antiviral, anti-inflammatory, and rich antioxidant properties (134). *Astragalus* polysaccharides were also found to improve growth performance, enhance the immune system, and stimulate disease resistance to Edwardsiellosis in catla (*Catla catla*). *Astragalus* polysaccharides are bioactive compounds that are reported to possess rich antioxidant properties (135) and can play important roles in activating the immune system (101). *Mitracarpus scaber* leaf extract was reported to stimulate disease resistance of African catfish against Edwardsiellosis (99). This plant leaf extract possesses bioactive compounds, such as eugenol and gallic acid. These bioactive compounds are able to modulate gut microbiota, enhance feed utilization, and promote growth performance (99). Green alga, *Chaetomorpha aerea* extract may carry bioactive compounds that can play a role as an activator for genes related to antimicrobial molecules production (98). Therefore, this green alga extract was found to stimulate disease resistance in *Labeo rohita* against Edwardsiellosis (98). Cassic acid is a bioactive compound widely and commercially used in Chinese herb medicinal (100). This compound can be found in the root and leaf of plant species, such as Senna, Rheum, and Cassia (100). Cassic acid has medicinal values like antibacterial, antifungal, and antiviral properties and is rich in antioxidant properties (69, 136). These medicinal values were responsible for the growth performance improvement and disease resistance to Edwardsiellosis in African catfish, *C. gariepinus* that received cassic acid as a feed additive (69). Brown alga, *E. cava* was found to be used as a prebiotic in promoting the growth of probiotic, LAB (32). Further study on *E. cava* revealed that brown alga can be used as a feed additive in zebrafish. It can improve the growth of zebrafish and stimulate disease resistance to Edwardsiellosis (105). A recent report showed that apple mangrove *Sonneratia caseolaris* extract could be used feed additive in African catfish, *C. gariepinus*. The bioactive compounds in the plant extract can enhance the appetite of the fish, improve growth performance, and stimulate disease resistance to Edwardsiellosis (102). Besides that, agricultural waste, a citrus by-product, was claimed to help in improving the health status of aquaculture species against Edwardsiellosis (103). For example, essential oil from *Citrus limon* carries bioactive compounds, such as flavonoid, coumarin, and limonene, that are responsible for the antibacterial, antioxidant, and anticancer properties of the essential oil (103). Bioactive compounds in the phytobiotics can play an important role in activating the innate immune system in aquatic animals (104). One of the innate immune systems is serum lysozyme. Serum lysozyme catalyzes the pathogen cell wall and phagocytosis activities against pathogens, such as viruses, parasites, and bacteria, that invade the host (104). All phytobiotics administered as a feed additive have been shown to improve the health status of aquatic animals against Edwardsiellosis except for nanoscale β-glucan (NSBG), which can also be used to fish larvae *via* bathing treatment (105). In this context, NSBG acted as an immunostimulant to enhance the innate immune system in the fish larval before the larval adaptive immune system was well developed (105). β-Glucan is a commercial feed additive that is abundant and inexpensive. Therefore, this bioactive compound is widely used in animal feed to enhance animal production. The duration of phytobiotics served as a feed additive to aquatic animal range from 14 to 91 days. The benefits of phytobiotics used as feed additives are not only to stimulate disease resistance to Edwardsiellosis but also to improve growth performance and enhance the immune system of aquatic animals. Administration of phytobiotics orally is the most practical and non-stressful method, and can be used widely in aquaculture.

## The adverse impacts of using phytobiotics in aquaculture

The application of phytobiotics in aquaculture was widely reported in the literature. Phytobiotics have beneficial effects on various aquatic animals, such as growth performance improvement, immune system enhancement, and disease resistance enhancement. Apart from their beneficial effects, phytobiotics were reported to have adverse impacts. For example, RVS has medicinal properties, such as anticancer (137), antiviral (138), antibacterial, and antioxidant (139) activities. This phytobiotic was reported to relieve the impacts of Edwardsiellosis infection in olive flounder (*P. olivaceus*) (33). In addition, the methanolic extract of RVS bark was also found to be significantly effective against *E. tarda* and *Vibrio anguillarum* (107). However, RVS possesses bioactive compounds known as urushiol congeners that can cause adverse effects, such as inflammation, blistering, and irritation (140). Thus, the adverse impacts limit the use of RSV in treatment. On the other hand, urushiol congeners were found absent in the RVS lignum (139). Furthermore, RVS lignum performed the highest antibacterial and antioxidant activities against *E. tarda* isolated from fish (141). Hence, RVS lignum has a high potential to be used as a phytobiotic in aquaculture. Some phytobiotics have low toxicity and few side effects on aquatic animals. For instance, *Astragalus* polysaccharides were widely used in Chinese medicine practice (101). These bioactive compounds were also shown to be promising as feed additives in chicken (142) and fish (101).

## Conclusion and recommendation

Edwardsiellosis due to *E. tarda* is an important disease in aquaculture that mainly affects carp fish, eels, flounder, turbot, channel catfish, and many other aquaculture species. This disease can devastate the whole fish farm and lead to huge economic loss. Traditionally, antibiotics and vaccines were used to combat Edwardsiellosis in aquaculture. However, antibiotics have an adverse impact on microbial communities in aquaculture sites, and their residues in aquaculture products can pose a threat to public health. On the other hand, the application of vaccines is expensive and requires high labor work. Therefore, these two issues became major constraints to the usage of vaccines in combating Edwardsiellosis in aquaculture. Therefore, phytobiotics can be an alternative option to fish farmers as a prophylactic agent against Edwardsiellosis in aquaculture. At present, phytobiotics are evidenced to have a high potential in controlling Edwardsiellosis. However, further studies should be carried out to investigate the effectiveness of phytobiotics against Edwardsiellosis in more important aquaculture species, such as eels, flounder, turbot, and channel catfish. Currently, there is a lack of information in the literature on the benefit of phytobiotics to the abovementioned aquaculture species, and many potential phytobiotics are waiting to be explored to relieve the impact of Edwardsiellosis in aquaculture.

## Author contributions

Conceptualization: KG and NZ. Writing—original draft preparation: ZA and LW. Writing—review and editing: MR, MK, NA, AK, GT-I, and AT. All authors have read and agreed to the published version of the manuscript.
